# The Anticancer Effect of Natural Plant Alkaloid Isoquinolines

**DOI:** 10.3390/ijms22041653

**Published:** 2021-02-06

**Authors:** Dahye Yun, So Young Yoon, Soo Jung Park, Yoon Jung Park

**Affiliations:** 1 System Health & Engineering Major in Graduate School (BK21 Plus Program), Ewha Womans University, Seoul 03760, Korea; dahye342@gmail.com (D.Y.); syyoon0504@gmail.com (S.Y.Y.); 2 Department of Nutritional Science and Food Management, Ewha Womans University, Seoul 03760, Korea; 3 Department of Sasang Constitutional Medicine, Woosuk University, Jeollabuk-do 55338, Korea

**Keywords:** isoquinoline alkaloids, anticancer, cell cycle arrest, apoptosis, autophagy, epigenetic regulation

## Abstract

Isoquinoline alkaloids-enriched herbal plants have been used as traditional folk medicine for their anti-inflammatory, antimicrobial, and analgesic effects. They induce cell cycle arrest, apoptosis, and autophagy, leading to cell death. While the molecular mechanisms of these effects are not fully understood, it has been suggested that binding to nucleic acids or proteins, enzyme inhibition, and epigenetic modulation by isoquinoline alkaloids may play a role in the effects. This review discusses recent evidence on the molecular mechanisms by which the isoquinoline alkaloids can be a therapeutic target of cancer treatment.

## 1. Introduction

Cancer is a leading cause of death worldwide and has a major impact on society. It is a major barrier to increasing life expectancy this century [1]. The World Health Organization (WHO) estimates that cancer was responsible for an estimated 9.6 million deaths in 2018 [2]. Treatment varies depending on the type and stage of cancer. Most people undergo a combination of treatments, such as surgery with chemotherapy and radiation therapy. However, adverse reactions to conventional treatment and drug resistance have led some to use complementary and alternative medicine (CAM) in conjunction with conventional medical treatments [3,4,5,6]. As interest in complementary therapies increases, so has the value of natural remedies [7]. Isoquinoline alkaloids, a group of plant-derived bioactive compounds, have traditionally been used as alternative treatments for their anti-inflammatory, antimicrobial, and analgesic effects [8,9,10,11,12]. Recently, biomedical and pharmacological developments have begun to uncover the anticancer effects and mechanisms of isoquinoline alkaloids. In this review, we discuss the anti-cancer effects and mechanisms of isoquinoline alkaloids.

## 2. Isoquinoline Alkaloids Derived from Various Herb Extracts

**Figure 1:**
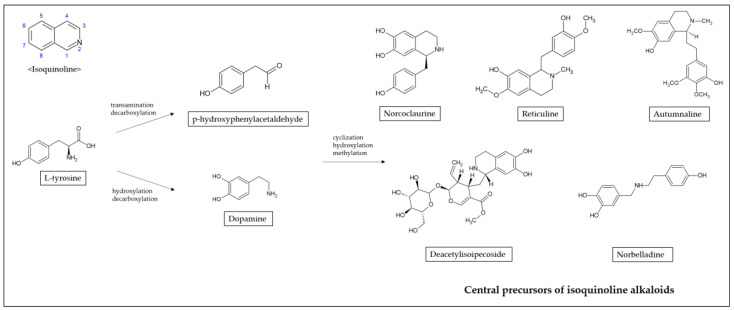
Synthesis of isoquinoline alkaloids.

Alkaloids that possess an isoquinoline moiety are one of the largest groups of natural substances. Isoquinoline is a heterocyclic compound consisting of a benzene and pyridine ring fused at C3/C4 of the pyridine ring [13]. The biosynthetic pathways of isoquinoline alkaloids proceed via tyrosine generating dopamine and *p*-hydroxyphenylacetaldehyde (Figure 1). Tyrosine is converted to dopamine by hydroxylation and decarboxylation, and to *p*-hydroxyphenylacetaldehyde by transamination and decarboxylation [14]. Through cyclization, hydroxylation, and methylation, dopamine and *p*-hydroxyphenylacetaldehyde are condensed to form specific scaffold molecules such as norcoclaurine, reticuline, autumnaline, deacetylisoipecoside, or norbelladine, central precursors to several thousand isoquinoline alkaloids [15,16]. 

Isoquinoline alkaloids have been used in folk medicine and have attracted attention in the pharmacological industry and among researchers due to their potential medicinal benefits. Most of the isoquinoline alkaloids discovered to date have been derived from plants, such as Alangiaceae, Annonaceae, Berberidaceae, Fabaceae, Fumariaceae, Lauraceae, Menispermaceae, Papaveraceae, Ranunculaceae, and Rutaceae [17]. Opium poppy (*Papaver somniferum*) is one of the oldest plant sources of commercial medicinal isoquinolines in the world. Morphine, codeine, papaverine, noscapine, and thebaine were detected in its latex [18], and more than 40 isoquinoline alkaloids have been isolated from opium [19]. *Chelidonium majus* L., of the Papaveraceae family, contains sanguinarine, chelidonine, chelerythrine, berberine, and coptisine [20]. 8-oxoberberine, berbidine, berbamine, aromoline, obamegine, berberine, and palmatine were obtained from *Berberis vulgaris* [21].

**Figure 2:**
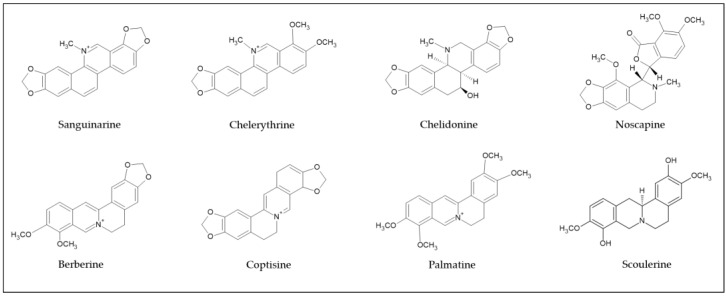
Examples of isoquinoline alkaloids’ structures.

Based on the structural diversity, isoquinoline alkaloids are classified into the subgroups benzylisoquinoline, aporphine, protoberberine, benzo[c]phenanthridine, protopine, phthalide isoquinoline, morphine, emetine, and pavine [17,22]. Berberine, palmatine, coralyne, and coptisine are the isoquinoline alkaloids from the protoberberine class, while sanguinarine, chelerythrine, and chelidonine are the main members of the benzo[c]phenanthridine class. Noscapine and scoulerine belong to the benzylisoquinoline alkaloid class. The most common examples of isoquinoline alkaloids (Figure 2) have been intensely investigated for their phytoceutical function.

## 3. Biological Functions

Isoquinoline alkaloids have various biochemical properties related to their binding to various differential biological functional ligands [23]. Isoquinoline alkaloids intercalate with polymorphic nucleic acid structures. Berberine and palmatine bind to B-form DNA and coralyne binds to duplex B-form DNA and a single-stranded poly(A) structure [24]. Spectroscopic and thermodynamic studies suggest that sanguinarine and berberine bind to the DNA and RNA double and triple helical structures [25] and sanguinarine binds to tRNAphe [26]. Interactions between sanguinarine and chelerythrine with DNA were both enthalpy- and entropy-favored actions [27]. 

Isoqinoline alkaloids inhibit the activity of some enzymes, especially acetylcholinesterase (AChE) and butylcholinesterase (BuChE) through anticholinesterase potency of alkaloid scaffolds [28,29,30,31,32,33,34]. This mechanism was uncovered via structure-based virtual screening [35]. Possible structure–activity relationship (SAR) investigations for active compounds predict that the protoberberine scaffold structure is associated with AChE inhibitory effects. Galanthamine from the Hippeastrum species inhibited the activity of AChE more than 90% compared to the control in the hippocampus of adult Wistar rats [28]. Chelidonine, 6-ethoxydihydrosanguinarine, and 6-ethoxydihydrochelerythrine, which are abundant in *Chelidonium majus* (Papaveraceae), exhibited inhibitory activity of human blood AChE and human plasma BuChE [36]. 

Protoberberine and coralyne are known as topoisomerase I and II inhibitors [29,30]. They exhibit intercalative and minor groove binding to duplex DNA and are involved in topoisomerase I poisoning [37]. In addition, corydine, parfumine, 8-methyl-2,3,10,11-tetraethoxyberbine, and chelidonine from the Papaveraceae family inhibit cytochrome P450 3A4 (CYP3A4) with high-affinity alkaloid interactions [31,32]. Berberine inhibited transcriptional activity of cyclooxygenase-2 (COX-2) through the binding to DNA and RNA. 

Isoquinoline alkaloids reportedly have other bioactivities, including antibacterial and antifungal effects via the binding to DNA and RNA [38,39,40]. (+)-*N*-(methoxycarbonyl)-*N*-nordicentrin, (+)-*N*-(methoxycarbonyl)-*N*-norpredicentrin, and (+)-*N*-(methoxycarbonyl)-*N*-norglaucine in the *L. cubeba* extract inhibited the bacterium *S. aureus* and fungus *A. alternata* and *C. nicotianae* [41]. Sanguinarine and chelerythrine from *Sanguinaria canadensis* and berberine and β-hydrastine from *Hydrastis canadensis* inhibited Staphylococcus aureus growth [42,43]. The antifungal activity of berberine and jatrorrhizine isolated from *Mahonia aquifolium* was evaluated against Malassezia [44]. Berberine inhibited the growth of H1N1 influenza A [45] and the Chikungunya virus [46]. 

Furthermore, isoquinoline alkaloids have anti-inflammatory and antioxidant effects. Berberine hydrochloride showed significantly low expression levels of inflammation markers and toll-like receptor 4 (TLR4) protein expression in lipopolysaccharide (LPS)-induced mice [47]. The downregulation of inflammatory cytokines such as TNFα, IL-6, and C-reactive protein by berberine treatment was confirmed in vitro [48]. Chelidonine, a major compound of *Chelidonium majus*, also inhibited LPS-induced inflammatory responses through TLR4/NF-κB signaling pathway suppression in RAW264.7 cells [49]. In a radical scavenging assay, iraqiine, muniranine, and kinabaline showed antioxidant activity [50], and stylopine, protopine, fumaritine, fumaricine, fumarophycine, fumariline, and fumarofine from two Algerian species of Fumaria inhibited lipid peroxidation [51]. 

## 4. Anticancer Effects of Isoquinoline Alkaloids

The anti-cancer activity of isoquinoline alkaloids is noteworthy. Isoquinoline alkaloids and/or isoquinoline-enriched plants have been investigated as alternative regimens to complement chemotherapy. They efficiently induce cell death in various cancer cell lines [52,53,54,55]. The evidence based on in vivo and in vitro models indicated isoquinoline alkaloids exert significant anti-cancer effects through cell cycle arrest, apoptosis, and autophagy (Table 1), leading to cell death. 

### 4.1. Apoptosis-Mediated Cell Death

Apoptosis, programmed cell death, is a promising target for anticancer therapy. Apoptosis is triggered by the extrinsic and intrinsic pathways. The extrinsic pathway is triggered by external stimuli. Ligand and death receptor (DR) binding interacts with the Fas-associated death domain (FADD) and tumor necrosis factor receptor 1 (TNFR1)-associated death domain (TRADD). A death-inducing signaling complex (DISC) is then formed and caspase-8 is recruited to DISC. This leads to the activation of caspase-8, which cleaves and activates caspase-3/6/7, initiating apoptosis [56].

The intrinsic pathway is triggered by exogenous and endogenous stimuli, including DNA damage and oxidative stress. The Bcl family members, Bax and Bcl-2, act as pro- or anti-apoptotic regulatory proteins through binding to the mitochondrial membrane. The release of cytochrome C in the cytoplasm recruits Apaf–1 and procaspase-9 to form the apoptosome, which triggers downstream caspase-9/3 cascades [57].

#### 4.1.1. Caspase-Dependent Apoptosis

Caspase activation is a central process for apoptosis. All caspases are produced as catalytically inactive zymogens and are cleaved and activated during apoptosis [58]. Chelerythrine-induced apoptosis was accompanied by a decrease in the mitochondrial membrane potential (MMP), the release of cytochrome c, activation of caspase-3 and poly ADP-ribose polymerase (PARP), and downregulation of Bcl-2 in BGC-823 cells [59]. Sanguinarine inhibited tumor growth in vivo and in vitro in various cancers, including prostate [60], cervical [61], pancreatic [62], and colorectal cancers [63]. AsPC-1 and BxPC-3 growth were suppressed via an increase in Bax, Bid, and Bak and decreases in the anti-apoptotic Bcl-2 and Bcl-xL proteins [62]. Sanguinarine also decreased the tumor size in orthotopical colorectal carcinoma bearing BALB/c-nu mice through increased caspase 3, PARP, and mitochondrial reactive oxygen species (ROS) cleavage [63]. The effect of chelerythrine on A549 and H1299 leads to increased protein levels of cleaved PARP and cleaved caspase 3 [64]. Chelidonine inhibited non-small cell lung cancer growth via regulating epidermal growth factor receptor/AMP-activated protein kinase (EGFR/AMPK) signaling pathways in vivo and in vitro [65]. Berberine induced caspase 3, 8, and 9 mediated apoptosis in A549 and H1299 xenograft mice models [66,67] and triple-negative breast cancer cells [68].

#### 4.1.2. MAPK-Mediated Apoptosis

Mitogen-activated protein kinase (MAPK) signaling pathways regulate fundamental cellular processes such as growth, proliferation, differentiation, and migration [69]. MAPK subfamilies consist of extracellular signal-regulated kinases (ERKs), c-Jun N-terminal kinases (JNKs), and p38-MAPKs. ERKs are important for cell survival, while JNKs and p38-MAPKs are stress-responsive and mediate apoptotic processes triggered by numerous stimuli [70]. The major cellular receptor protein kinase C (PKC) activates the MAPK/ERK pathway via c-Raf [71]. Berberine treatment of A549 cells showed indication of apoptosis with increased phosphorylation of p38-MAPK and induced protein expression of p53 and forkhead box class O 3a (FOXO3a) [72]. Berberine affected PKC, glycogen synthase kinase 3 beta (GSK-3β), ERK activity, and (NSAID) activated gene-1 (NAG-1) expression, resulting in apoptosis in HCT-116 cells [73]. 

### 4.2. Cell Cycle Arrest

The cell cycle is regulated by several cyclin-dependent kinases and controls cell division and proliferation. Induction of cell cycle arrest and inhibition of cell proliferation by regulation of cell cycle checkpoints is a therapeutic target for treating cancer [74]. Berberine leads to G1 cell cycle arrest with the induction of NAG1 and activating transcription factor 3 (ATF3) expression on HCT116 cells [73]. An antitumor effect has been demonstrated in human colorectal adenocarcinoma by inducing G2/M phase arrest in vivo and in vitro studies [75]. Berberine treatment also caused G2 phase arrest in U251 cells and significantly inhibited tumor progression in the glioma mouse model [76]. Chelerythrine treatment induced S phase arrest to inhibit BGC-823 cell proliferation [59]. Moreover, sanguinarine arrested AsPC-1 and BXPC-3 cells in the G0–G1 phase through modulation of the Bcl-2 family [62]. 

### 4.3. Autophagy-Mediated Cell Death

Autophagy is a response to a range of cellular stressors to maintain cellular homeostasis. Therefore, autophagy is a critical mechanism of cancer treatments. Mechanistic target of rapamycin (mTOR), a molecular regulator of autophagy, is associated with cell proliferation and is regulated by AMPK. Inhibition of mTORC1 and increased AMPK induces autophagy [77], during which autophagosomes are formed to digest cytoplasmic components and LC3I is converted to LC3II [78,79]. Berberine upregulated LC3-II and induced autophagy in glioblastoma through the regulation of the AMPK/mTOR/unc-51 like autophagy activating kinase 1 (ULK1)-pathway [80] and repressed human gastric cancer cell proliferation through inactivation of the MAPK/mTOR/p70S6K/Akt signaling pathway in vivo and in vitro [81]. In chelerythrine-treated A549 and H1299 cells, LC3-II expression was enhanced [64]. Similarly, neferine upregulated LC3-II and downregulated the phosphoinositide 3-kinase (P13K), Akt, and mTOR pathways, inducing autophagy [82].

**Table 1:** Current evidence on anticancer effects of isoquinoline alkaloids.

Mechanisms	Cancer Type	Effect	Compounds	Reference
Apoptosis	Colorectal cancer	Accumulation of cells in sub G0 phase Increase in Bax expression	Berberine	[73]
	Breast cancer	Condensed chromatin with fragmented nuclei Accumulation of cells in sub G0 phase	Noscapine	[83]
	Gastric cancer	Decrease of mitochondrial membrane potential Increased release of cytochrome c Activation of caspase-3/8/9 and PARP Decrease in Bcl-2 expression Increase in Bax expression Apoptotic DNA fragmentation	Chelerythrine	[59]
	Breast cancer Liver cancer Lung cancer Prostate cancer		Berberine	[66,67,68,84,85,86]
	Leukemia		Berberine Scoulerine	[87,88]
	Colorectal cancer		Noscapine Sanguinarine	[41,63]
	Breast cancer	Decrease of mitochondrial membrane potential Increased phosphorylation of JNK Increased release of cytochrome c and AIF Activation of caspase-3 Decrease in Bcl-2 expression Increase in Bax expression	Berberine	[89]
	Colorectal cancer		Liensinine	[90]
	Lung cancer	Increased phosphorylation of p38 MAPK Increase in transcriptional activity of FoxO3a	Berberine	[72]
	Liver cancer	Suppressed PI3K/Akt/mTOR pathway Increased phosphorylation of JNK Reactive oxygen species (ROS) generation Increase in Bim expression and transcriptional activity of FoxO	Berberine	[91]
	Prostate cancer	Decrease of mitochondrial membrane potential Decrease in Bcl-2, Bcl-XL, and XIAP expression Increase in Bax, Bad, and Apaf-1 expression Increased cytochrome c and AIF release Activation of caspase-3 and PARP Suppression of PI3K/Akt pathway	Sinomenine	[92]
	Liver cancer		Tetrandrine	[93]
	Lung cancer	Reactive oxygen species (ROS) generation Activation of caspase-3/8/9 and PARP Endoplasmic reticulum (ER) stress activation Increased phosphorylation of JNK Suppression of PI3K/Akt pathway	Chelerythrine	[64]
	Liver cancer Colorectal cancer		Coptisine	[94,95]
	Colorectal cancer		Scoulerine	[96]
	Renal cancer	Decreased phosphorylation of ERK and Akt Decrease in Bcl-2 expression Increase in Bax and p53 expression	Chelerythrine	[97]
	Oral cancer	Increase in FasL expression Decrease in Bcl-2 and Bcl-xL expression Increase in Bax, Bad, and Apaf-1 expression Activation of caspase-3/8/9 and PARP Increased phosphorylation of p38 MAPK	Berberine	[98]
Cell cycle arrest	Breast cancer Colorectal cancer Gastric cancer Pancreatic cancer Prostate cancer	G1 phase cell cycle arrest	Berberine Sanguinarine Chelerythrine	[57,60,61,86,99]
	Colorectal cancer Glioblastoma Lung cancer	G1 phase cell cycle arrest induction of p21 inhibition of cyclin D1	Tetrandrine Berberine	[59,76,100,101]
	Gastric cancer Ovarian cancer	S phase cell cycle arrest	Chelerythrine Liriodenine	[59]
	Glioblastoma	G2/M phase cell arrest Enhanced cyclin dependent kinase 1 (Cdk1)/cyclin B1 complex activity	Chelidonine	[102]
	Colorectal cancer		Liensinine Noscapine Berberine	[41,58,92]
	Leukemia		Scoulerine	[88]
	Breast cancer		Noscapine	[83]
	Prostate cancer		Protopine	[103]
Autophagy	Breast cancer Gastric cancer Glioblastoma cancer Liver cancer Lungcancer	Enhanced expression of LC3-II Increase of AMPK activity Downregulated expression of PI3K, Akt, and mTOR Activation of Beclin-1	Berberine Neferine Sanguinarine Chelerythrine	[64,80,81,82,86,99,100]

## 5. Molecular Mechanisms of Anticancer Effects

The molecular or cellular mechanisms behind these anti-cancer effects are of great interest. Molecular functions, such as binding to nucleic acids or proteins and enzyme inhibition, have been suggested as potential anti-cancer mechanisms.

### 5.1. Binding to Polynucleic Acids

Interactions of the alkaloids with DNA and RNA may be responsible for anticancer effects. Specific binding to nucleic acids regulates polynucleic acid stability and may be the therapeutic target of isoquinoline alkaloids with anticancer effects. These bindings disrupt the structure of duplex B-form DNA and affect their interaction with DNA replication, repair, or transcription-related proteins. Sanguinarine and chelerythrine preferred double-helical regions for binding [27] and DNA adduct formed from both isoquinoline alkaloids [101].

### 5.2. Binding to Microtubules

Microtubule polymerization plays a pivotal role in chromosomal segregation during mitosis [104]. Specific binding to mitotic microtubules has been considered the therapeutic target of isoquinoline alkaloids with anticancer effects. Sanguinarine caused microtubule depolymerization and conformational changes in tubulin through tubulin binding and inhibited cell proliferation in Hela cells [105]. Noscapine-treated MCF-7, MDA-MB-231, and CEM cells displayed higher tubulin-binding activity and mitotic arrest followed by apoptosis [83,106]. Chelidonine [107] and hydroxy-substituted indolo[2,1-a]isoquinolines [108] disrupt microtubular structure and inhibit tubulin polymerization.

### 5.3. Inhibition of Enzyme Activity

Inhibition of enzyme activity is associated with anticancer activities. The abilities of protoberberine and coralyne as topoisomerase I and II inhibitors are well known [29,30]. Berberrubine’s inhibition of DNA topoisomerase II induced DNA cleavage through stabilization of the enzyme–DNA complexes [109,110].

Telomere shortening is evident in MCF-7 cells upon chelidonine treatment [111]. A new berberine derivative synthesized telomeric quadruplex ligands and led to inhibitory effects on telomerase activity [112,113]. Berberine also downregulates nucleophosmin/B23 and inhibits telomerase activity and induces apoptosis of HL-60 cells [114].

Corydine, parfumine, 8-methyl-2,3,10,11-tetraethoxyberbine, and chelidonine from the Papaveraceae family inhibit CYP3A4, indicating a high-affinity interaction with this enzyme and demonstrating an anticancer effect [31,32]. The binding of chelerythrine to Bcl-2 and apoptotic processes were observed in a dose-dependent manner [115,116]. Berberine inhibited cyclooxygenase-2 (COX-2) transcriptional activity with the regulation of I kappa B kinase (IKK) and nuclear factor-kappa B (NF-κB), and induced apoptosis [33,34]. However, inhibition of AChE and BuChE activity is not related to anticancer effects. Studies have shown that AChE is upregulated in response to apoptotic induction [117]. Its inhibition is considered a potential treatment of Alzheimer’s disease (AD). AD is characterized by a loss of neurotransmission due to abnormal synaptic acetylcholine levels [118]. AChE and BuChE are enzymes that break down the neurotransmitter acetylcholine and regulate cholinergic levels in the brain [119].

### 5.4. Epigenetic Modulation

Epigenetics is defined as the heritable changes in gene expression without alteration of the DNA sequence itself [120]. Epigenetic dysregulation of gene expression occurs during stages of cell proliferation, invasion, metastasis, and cancer development [121,122,123]. DNA methylation and histone modifications, as main epigenetic mechanisms, induce chromatin remodeling followed by changes in cellular phenotypes [124]. These mechanisms regulate proto-oncogene, tumor suppressor gene, and DNA repair gene expression.

Natural products including the secondary metabolites found in plants are reported to reverse cancer progression through modulation of epigenetic events, such as modulation of the activities of DNA methyltransferases (DNMTs) and histone deacetylases (HDACs) [125,126]. Remarkably, isoquinoline alkaloids act as putative targets in cancer drug development by affecting epigenetic modulation (Table 2).

Particularly, berberine’s anticancer effects have been associated with DNA and histone modifications [127,128,129,130]. In berberine-treated HepG2 cells, inhibition of DNA methylation in promoter regions of the cytochrome P450 2B6 (CYP2B6) and CYP3A4 genes mediated an anti-proliferative effect [127]. In U266 cells, berberine induced apoptosis by suppression of NF-κB nuclear translocation through Set9-mediated lysine methylation and decreased miR21 levels [129]. Treatment with berberine affected DNMT1, DNMT3A, DNMT3B, miR-152, miR-429, and miR-29a expression, which are critical regulators of colon cancer initiation and progression [130]. Berberine also repressed HDAC activity and triggered sub-G0/G1 cell cycle arrest in A549 cells [128]. Sanguinarine inhibited H3K9, H3K4, and H3R17 methylation in vivo and in vitro [131].

**Table 2:** Epigenetic modulation in isoquinoline-induced cell death.

Tumor Type	Compounds	Effect	Reference
Liver cancer	Berberine	Reduced DNA methylation level in promoter regions of CYP2B6 and CYP3A4 genes	[127]
Myeloma	Berberine	Increased the level of Set9 (lysine methyltransferase) Increased the level of methylation of the RelA subunit Inhibited NF-κB nuclear translocation and miR-21 transcription Hypomethylation of p53 promoter	[129,132]
Colorectal cancer	Berberine	Increased the level of DNMT1, DNMT3A, DNMT3B Increased the level of miR-152, miR-429, miR-29a	[130]
Lung cancer	Berberine	Decrease of HDAC activity Hyperacetylated histones H3 and H4 Decreased level of tumor necrosis factor-α (TNF-α), COX-2, MMP-2, and MMP-9 Increased the level of p21 and p53	[128]
Cervical cancer	Sanguinarine	Reduced H3K9, H3K4, and H3R17 methylation	[131]

## 6. Conclusions

**Figure 3:**
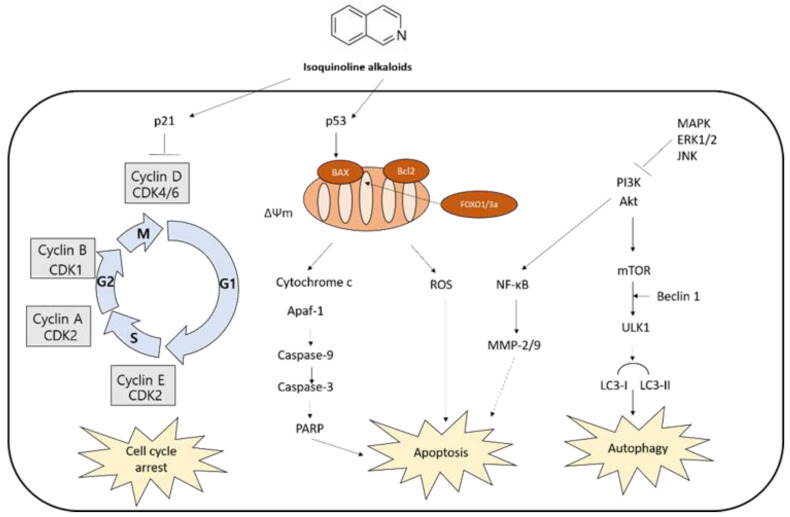
Molecular pathways involved in anticancer mechanisms.

Current evidence demonstrates that isoquinoline alkaloids have anticancer effects such as induction of cell cycle arrest, apoptosis, and autophagy (Figure 3), suggesting their potential as a cancer therapeutic agent. The effects are, at least in part, attributed to their binding to DNA or proteins, inhibition of enzyme activity, or epigenetic modulation. Further studies are needed to fully discover the underlying mechanisms of isoquinoline alkaloid-mediated cell death against cancer.

## Abbreviation

AChE	Acetylcholinesterase
AD	Alzheimer’s disease
AMPK	AMP-activated protein kinase
ATF3	Activating transcription factor 3
BuChE	Butylcholinesterase
CAM	Complementary and alternative medicine
CDK1	Cyclin Dependent Kinase 1
COX-2	Cyclooxygenase-2
CYP2B6	Cytochrome P450 2B6
CYP3A4	Cytochrome P450 3A4
DISC	Death-inducing signaling complex
DNMT	DNA methyltransferase
DR	Death receptor
EGFR	Epidermal growth factor receptor
ERK	Extracellular signal-regulated kinase
FADD	Fas-associated death domain
FOXO3a	Forkhead box class O 3a
GSK-3β	Glycogen synthase kinase 3 beta
HDAC	Histone deacetylase
IKK	I kappa B kinase
JNK	Jun N-terminal kinase
LPS	Lipopolysaccharide
MAPK	Mitogen-activated protein kinase
mTOR	Mechanistic target of rapamycin
NAG-1	(NSAID) activated gene-1
NF-κB	Nuclear factor-kappa B
PARP	Poly ADP-ribose polymerase
PI3K	Phosphoinositide 3-kinase
PKC	Protein kinase C
ROS	Reactive oxygen species
SAR	Structure–activity relationship
TLR4	Toll-like receptor 4
TNFR1	Tumor necrosis factor receptor 1
TNF-α	Tumor necrosis factor-α
TRADD	TNFR1-associated death domain protein
ULK1	Unc-51 Like Autophagy Activating Kinase 1

## References

[1] Sener S.F., Grey N. (2005). The global burden of cancer. J. Surg. Oncol..

[2] Bray F., Ferlay J., Soerjomataram I., Siegel R.L., Torre L.A., Jemal A. (2018). Global cancer statistics 2018: GLOBOCAN estimates of incidence and mortality worldwide for 36 cancers in 185 countries. CA Cancer J. Clin..

[3] Deng G., Cassileth B. (2013). Complementary or alternative medicine in cancer care—myths and realities. Nat. Rev. Clin. Oncol..

[4] Jermini M., Dubois J., Rodondi P.-Y., Zaman K., Buclin T., Csajka C., Orcurto A., Rothuizen L.E. (2019). Complementary medicine use during cancer treatment and potential herb-drug interactions from a cross-sectional study in an academic centre. Sci. Rep..

[5] Choi B.Y., Joo J.-C., Lee Y.K., Jang I.-S., Park S.J., Park Y.J. (2017). Anti-cancer effect of Scutellaria baicalensis in combination with cisplatin in human ovarian cancer cell. BMC Complement. Altern. Med..

[6] Lee Y.K., Lim J., Yoon S.Y., Joo J.-C., Park S.J., Park Y.J. (2019). Promotion of Cell Death in Cisplatin-Resistant Ovarian Cancer Cells through KDM1B-DCLRE1B Modulation. Int. J. Mol. Sci..

[7] Mukherjee A., Basu S., Sarkar N., Ghosh A. (2001). Advances in cancer therapy with plant based natural products. Curr. Med. Chem..

[8] Gerenčer M., Turecek P.L., Kistner O., Mitterer A., Savidis-Dacho H., Barrett N.P. (2006). In vitro and in vivo anti-retroviral activity of the substance purified from the aqueous extract of *Chelidonium majus* L.. Antivir. Res..

[9] Yang G., Lee K., Lee M.-H., Kim S.-H., Ham I., Choi H.-Y. (2011). Inhibitory effects of *Chelidonium majus* extract on atopic dermatitis-like skin lesions in NC/Nga mice. J. Ethnopharmacol..

[10] Biswas S.J., Bhattacharjee N., Khuda-Bukhsh A.R. (2008). Efficacy of a plant extract (*Chelidonium majus* L.) in combating induced hepatocarcinogenesis in mice. Food Chem. Toxicol..

[11] Nadova S., Miadokova E., Alfoldiova L., Kopaskova M., Hasplova K., Hudecova A., Vaculcikova D., Gregan F., Cipak L. (2008). Potential antioxidant activity, cytotoxic and apoptosis-inducing effects of *Chelidonium majus* L. extract on leukemia cells. Neuro Endocrinol. Lett..

[12] Mikołajczak P.Ł., Kedzia B., Ożarowski M., Kujawski R., Bogacz A., Bartkowiak-Wieczorek J., Białas W., Gryszczyńska A., Buchwald W., Szulc M. (2015). Evaluation of anti-inflammatory and analgesic activities of extracts from herb of *Chelidonium majus* L.. Cent. Eur. J. Immunol..

[13] Satyajit D., Lutfun N. (2007). Chemistry for Pharmacy Students General, Organic and Natural Product Chemistry.

[14] Chrzanowska M., Grajewska A., Rozwadowska M.D. (2016). Asymmetric synthesis of isoquinoline alkaloids: 2004–2015. Chem. Rev..

[15] Diamond A., Desgagné-Penix I. (2016). Metabolic engineering for the production of plant isoquinoline alkaloids. Plant Biotechnol. J..

[16] Civjan N. (2012). Natural Products in Chemical Biology.

[17] Brahmachari G. (2015). Bioactive Natural Products.

[18] Weid M., Ziegler J., Kutchan T.M. (2004). The roles of latex and the vascular bundle in morphine biosynthesis in the opium poppy, Papaver somniferum. Proc. Natl. Acad. Sci. USA.

[19] Preininger V. (1985). Chemotaxonomy of the Papaveraceae Alkaloids. The Chemistry and Biology of Isoquinoline Alkaloids.

[20] Sárközi Á., Janicsák G., Kursinszki L., Kéry Á. (2006). Alkaloid composition of *Chelidonium majus* L. studied by different chromatographic techniques. Chromatographia.

[21] Hostalkova A., Marikova J., Opletal L., Korabecny J., Hulcova D., Kunes J., Novakova L., Perez D.I., Jun D., Kucera T. (2019). Isoquinoline alkaloids from Berberis vulgaris as potential lead compounds for the treatment of Alzheimer’s disease. J. Nat. Prod..

[22] Steglich W., Fugmann B., Lang-Fugmann S. (2000). Römpp Encyclopedia Natural Products.

[23] Bhadra K., Kumar G.S. (2011). Therapeutic potential of nucleic acid-binding isoquinoline alkaloids: Binding aspects and implications for drug design. Med. Res. Rev..

[24] Maiti M., Kumar G.S. (2010). Polymorphic nucleic acid binding of bioactive isoquinoline alkaloids and their role in cancer. J. Nucleic Acids.

[25] Das S., Kumar G.S., Ray A., Maiti M. (2003). Spectroscopic and thermodynamic studies on the binding of sanguinarine and berberine to triple and double helical DNA and RNA structures. J. Biomol. Struct. Dyn..

[26] Hossain M., Kabir A., Suresh Kumar G. (2012). Binding of the anticancer alkaloid sanguinarine with tRNAphe: Spectroscopic and calorimetric studies. J. Biomol. Struct. Dyn..

[27] Basu P., Suresh Kumar G. (2015). A comparative study on the interaction of the putative anticancer alkaloids, sanguinarine and chelerythrine, with single-and double-stranded, and heat-denatured DNAs. J. Biomol. Struct. Dyn..

[28] Pagliosa L., Monteiro S., Silva K., De Andrade J., Dutilh J., Bastida J., Cammarota M., Zuanazzi J. (2010). Effect of isoquinoline alkaloids from two Hippeastrum species on in vitro acetylcholinesterase activity. Phytomedicine.

[29] Sanders M., Liu A., Li T.-K., Wu H.-Y., Desai S., Mao Y., Rubin E., Lavoie E., Makhey D., Liu L.F. (1998). Selective cytotoxicity of topoisomerase-directed protoberberines against glioblastoma cells. Biochem. Pharmacol..

[30] Gatto B., Sanders M.M., Yu C., Wu H.Y., Makhey D., Lavoie E.J., Liu L.F. (1996). Identification of topoisomerase I as the cytotoxic target of the protoberberine alkaloid coralyne. Cancer Res..

[31] Salminen K.A., Meyer A., Jerabkova L., Korhonen L.E., Rahnasto M., Juvonen R.O., Imming P., Raunio H. (2011). Inhibition of human drug metabolizing cytochrome P450 enzymes by plant isoquinoline alkaloids. Phytomedicine.

[32] El-Readi M.Z., Eid S., Ashour M.L., Tahrani A., Wink M. (2013). Modulation of multidrug resistance in cancer cells by chelidonine and *Chelidonium majus* alkaloids. Phytomedicine.

[33] Pandey M.K., Sung B., Kunnumakkara A.B., Sethi G., Chaturvedi M.M., Aggarwal B.B. (2008). Berberine modifies cysteine 179 of IκBα kinase, suppresses nuclear factor-κB–regulated antiapoptotic gene products, and potentiates apoptosis. Cancer Res..

[34] Fukuda K., Hibiya Y., Mutoh M., Koshiji M., Akao S., Fujiwara H. (1999). Inhibition by berberine of cyclooxygenase-2 transcriptional activity in human colon cancer cells. J. Ethnopharmacol..

[35] Dighe S.N., Deora G.S., De La Mora E., Nachon F., Chan S., Parat M.-O., Brazzolotto X., Ross B.P. (2016). Discovery and structure–activity relationships of a highly selective butyrylcholinesterase inhibitor by structure-based virtual screening. J. Med. Chem..

[36] Cahlíková L., Opletal L., Kurfürst M., Macáková K., Kulhánková A., Hošt’álková A. (2010). Acetylcholinesterase and butyrylcholinesterase inhibitory compounds from *Chelidonium majus* (Papaveraceae). Nat. Prod. Commun..

[37] Pilch D.S., Yu C., Makhey D., Lavoie E.J., Srinivasan A.R., Olson W.K., Sauers R.R., Breslauer K.J., Geacintov N.E., Liu L.F. (1997). Minor groove-directed and intercalative ligand− DNA interactions in the poisoning of human DNA topoisomerase I by protoberberine analogs. Biochemistry.

[38] Jin J., Hua G., Meng Z., Gao P. (2010). Antibacterial mechanisms of berberine and reasons for little resistance of bacteria. Chin. Herb. Med..

[39] Tan G.T., Pezzuto J.M., Kinghorn A.D., Hughes S.H. (1991). Evaluation of natural products as inhibitors of human immunodeficiency virus type 1 (HIV-1) reverse transcriptase. J. Nat. Prod..

[40] Sethi M.L. (1983). Enzyme inhibition VI: Inhibition of reverse transcriptase activity by protoberberine alkaloids and structure–activity relationships. J. Pharm. Sci..

[41] Yang Z.-R., Liu M., Peng X.-L., Lei X.-F., Zhang J.-X., Dong W.-G. (2012). Noscapine induces mitochondria-mediated apoptosis in human colon cancer cells in vivo and in vitro. Biochem. Biophys. Res. Commun..

[42] Gottshall R.Y., Lucas E.H., Lickfeldt A., Roberts J.M. (1949). The occurrence of antibacterial substances active against Mycobacterium tuberculosis in seed plants. J. Clin. Investig..

[43] Dermarderosian A. (1977). Medicinal teas-boon or bane. Drug Ther..

[44] Vollekova A., Košťálová D., Sochorova R. (2001). Isoquinoline alkaloids from *Mahonia aquifolium* stem bark are active against *Malassezia* spp.. Folia Microbiol..

[45] Cecil C.E., Davis J.M., Cech N.B., Laster S.M. (2011). Inhibition of H1N1 influenza A virus growth and induction of inflammatory mediators by the isoquinoline alkaloid berberine and extracts of goldenseal (Hydrastis canadensis). Int. Immunopharmacol..

[46] Varghese F.S., Thaa B., Amrun S.N., Simarmata D., Rausalu K., Nyman T.A., Merits A., McInerney G.M., Ng L.F.P., Ahola T. (2016). The antiviral alkaloid berberine reduces chikungunya virus-induced mitogen-activated protein kinase signaling. J. Virol..

[47] Wang X., Feng S., Ding N., He Y., Li C., Li M., Ding X., Ding H., Li J., Wu J. (2018). Anti-inflammatory effects of berberine hydrochloride in an LPS-induced murine model of mastitis. Evid. Based Complement. Altern. Med..

[48] Choi B.-H., Ahn I.-S., Kim Y.-H., Park J.-W., Lee S.-Y., Hyun C.-K., Do M.-S. (2006). Berberine reduces the expression of adipogenic enzymes and inflammatory molecules of 3T3-L1 adipocyte. Exp. Mol. Med..

[49] Liao W., He X., Yi Z., Xiang W., Ding Y. (2018). Chelidonine suppresses LPS-Induced production of inflammatory mediators through the inhibitory of the TLR4/NF-κB signaling pathway in RAW264. 7 macrophages. Biomed. Pharmacother..

[50] Aldulaimi A.K.O., Abd-Azziz S.S.S., Bakri Y.M., Nafiah M.A., Aowda S., Awang K., Litaudon M. (2019). Two New isoquinoline alkaloids from the bark of Alphonsea cylindrica King and their antioxidant activity. Phytochem. Lett..

[51] Maiza-benabdesselam F., Khentache S., Bougoffa K., Chibane M., Adach S., Chapeleur Y., Max H., Laurain-Mattar D. (2007). Antioxidant activities of alkaloid extracts of two Algerian species of Fumaria: Fumaria capreolata and Fumaria bastardii. Biol. Chem..

[52] Havelek R., Seifrtova M., Královec K., Krocova E., Tejkalova V., Novotny I., Cahlíková L., Safratova M., Opletal L., Bilkova Z. (2016). Comparative cytotoxicity of chelidonine and homochelidonine, the dimethoxy analogues isolated from *Chelidonium majus* L.(Papaveraceae), against human leukemic and lung carcinoma cells. Phytomedicine.

[53] Choi S.U., Baek N.-L., Kim S.-H., Yang J.H., Eun J.S., Shin T.Y., Lim J.P., Lee J.H., Jeon H., Yun M.-Y. (2007). Cytotoxic isoquinoline alkaloids from the aerial parts ofCorydalis incisa. Arch. Pharmacal Res..

[54] Al-ghazzawi A.M. (2019). Anti-cancer activity of new benzyl isoquinoline alkaloid from Saudi plant Annona squamosa. BMC Chem..

[55] Iizuka N. (2000). Inhibitory effect of Coptidis Rhizoma and berberine on the proliferation of human esophageal cancer cell lines. Cancer Lett..

[56] Elmore S. (2007). Apoptosis: A review of programmed cell death. Toxicol. Pathol..

[57] Fulda S., Debatin K.M. (2006). Extrinsic versus intrinsic apoptosis pathways in anticancer chemotherapy. Oncogene.

[58] Shi Y. (2002). Mechanisms of caspase activation and inhibition during apoptosis. Mol. Cell.

[59] Zhang Z., Guo Y., Zhang L., Zhang J., Wei X. (2012). Chelerythrine chloride from Macleaya cordata induces growth inhibition and apoptosis in human gastric cancer BGC-823 cells. Acta Pharm. Sin. B.

[60] Sun M., Lou W., Chun J.Y., Cho D.S., Nadiminty N., Evans C.P., Chen J., Yue J., Zhou Q., Gao A.C. (2010). Sanguinarine suppresses prostate tumor growth and inhibits survivin expression. Genes Cancer.

[61] Zhang H., Zhang J., Venkat P.S., Gu C., Meng Y. (2019). Sanguinarine exhibits potent efficacy against cervical cancer cells through inhibiting the STAT3 pathway in vitro and in vivo. Cancer Manag. Res..

[62] Ahsan H., Reagan-Shaw S., Breur J., Ahmad N. (2007). Sanguinarine induces apoptosis of human pancreatic carcinoma AsPC-1 and BxPC-3 cells via modulations in Bcl-2 family proteins. Cancer Lett..

[63] Gong X., Chen Z., Han Q., Chen C., Jing L., Liu Y., Zhao L., Yao X., Sun X. (2018). Sanguinarine triggers intrinsic apoptosis to suppress colorectal cancer growth through disassociation between STRAP and MELK. BMC Cancer.

[64] Tang Z.-H., Cao W.-X., Wang Z.-Y., Lu J.-H., Liu B., Chen X., Lu J.-J. (2017). Induction of reactive oxygen species-stimulated distinctive autophagy by chelerythrine in non-small cell lung cancer cells. Redox Biol..

[65] Xie Y.-J., Gao W.-N., Wu Q.-B., Yao X.-J., Jiang Z.-B., Wang Y.-W., Wang W.-J., Li W., Hussain S., Liu L. (2020). Chelidonine Selectively Inhibits the Growth of Gefitinib-resistant Non-small Cell Lung Cancer Cells through the EGFR-AMPK Pathway. Pharmacol. Res..

[66] Xiao Y., Tian C., Huang T., Han B., Wang M., Ma H., Li Z., Ye X., Li X. (2018). 8-Cetylberberine inhibits growth of lung cancer in vitro and in vivo. Life Sci..

[67] Katiyar S.K., Meeran S.M., Katiyar N., Akhtar S. (2009). p53 cooperates berberine-induced growth inhibition and apoptosis of non-small cell human lung cancer cells in vitro and tumor xenograft growth in vivo. Mol. Carcinog..

[68] Zhao Y., Jing Z., Lv J., Zhang Z., Lin J., Cao X., Zhao Z., Liu P., Mao W. (2017). Berberine activates caspase-9/cytochrome c-mediated apoptosis to suppress triple-negative breast cancer cells in vitro and in vivo. Biomed. Pharmacother..

[69] Seger R., Krebs E.G. (1995). The MAPK signaling cascade. FASEB J..

[70] Wada T., Penninger J.M. (2004). Mitogen-activated protein kinases in apoptosis regulation. Oncogene.

[71] Schönwasser D.C., Marais R.M., Marshall C.J., Parker P.J. (1998). Activation of the mitogen-activated protein kinase/extracellular signal-regulated kinase pathway by conventional, novel, and atypical protein kinase C isotypes. Mol. Cell Biol..

[72] Zheng F., Tang Q., Wu J., Zhao S., Liang Z., Li L., Wu W., Hann S.S. (2014). p38α MAPK-mediated induction and interaction of FOXO3a and p53 contribute to the inhibited-growth and induced-apoptosis of human lung adenocarcinoma cells by berberine. J. Exp. Clin. Cancer Res..

[73] Piyanuch R., Sukhthankar M., Wandee G., Baek S.J. (2007). Berberine, a natural isoquinoline alkaloid, induces NAG-1 and ATF3 expression in human colorectal cancer cells. Cancer lett..

[74] Otto T., Sicinski P. (2017). Cell cycle proteins as promising targets in cancer therapy. Nat. Rev. Cancer.

[75] Cai Y.C., Xia Q., Luo R.Z., Huang P.Y., Sun Y.L., Shi Y.X., Jiang W.Q. (2014). Berberine inhibits the growth of human colorectal adenocarcinoma in vitro and in vivo. J. Nat. Med..

[76] Liu Z., Chen Y., Gao H., Xu W., Zhang C., Lai J., Liu X., Huang H. (2020). Berberine Inhibits Cell Proliferation by Interfering with Wild-Type and Mutant P53 in Human Glioma Cells. OncoTargets Ther..

[77] Kim J., Kundu M., Viollet B., Guan K.-L. (2011). AMPK and mTOR regulate autophagy through direct phosphorylation of Ulk1. Nat. Cell Biol..

[78] Tanida I., Ueno T., Kominami E. (2008). LC3 and Autophagy. Methods Mol. Biol..

[79] Mah L.Y., Ryan K.M. (2012). Autophagy and cancer. Cold Spring Harb Perspect Biol..

[80] Wang J., Qi Q., Feng Z., Zhang X., Huang B., Chen A., Prestegarden L., Li X., Wang J. (2016). Berberine induces autophagy in glioblastoma by targeting the AMPK/mTOR/ULK1-pathway. Oncotarget.

[81] Zhang Q., Wang X., Cao S., Sun Y., He X., Jiang B., Yu Y., Duan J., Qiu F., Kang N. (2020). Berberine represses human gastric cancer cell growth in vitro and in vivo by inducing cytostatic autophagy via inhibition of MAPK/mTOR/p70S6K and Akt signaling pathways. Biomed. Pharmacother..

[82] Poornima P., Weng C.F., Padma V.V. (2013). Neferine from Nelumbo nucifera induces autophagy through the inhibition of PI3K/Akt/mTOR pathway and ROS hyper generation in A549 cells. Food Chem..

[83] Aneja R., Vangapandu S.N., Lopus M., Viswesarappa V.G., Dhiman N., Verma A., Chandra R., Panda D., Joshi H.C. (2006). Synthesis of microtubule-interfering halogenated noscapine analogs that perturb mitosis in cancer cells followed by cell death. Biochem. Pharmacol..

[84] Meeran S.M., Katiyar S., Katiyar S.K. (2008). Berberine-induced apoptosis in human prostate cancer cells is initiated by reactive oxygen species generation. Toxicol. Appl. Pharmacol..

[85] Mantena S.K., Sharma S.D., Katiyar S.K. (2006). Berberine, a natural product, induces G1-phase cell cycle arrest and caspase-3-dependent apoptosis in human prostate carcinoma cells. Mol. Cancer Ther..

[86] Wang N., Feng Y., Zhu M., Tsang C.-M., Man K., Tong Y., Tsao S.-W. (2010). Berberine induces autophagic cell death and mitochondrial apoptosis in liver cancer cells: The cellular mechanism. J. Cell. Biochem..

[87] Jantova S., Cipak L., Letasiova S. (2007). Berberine induces apoptosis through a mitochondrial/caspase pathway in human promonocytic U937 cells. Toxicol. In Vitro.

[88] Habartova K., Havelek R., Seifrtova M., Kralovec K., Cahlikova L., Chlebek J., Cermakova E., Mazankova N., Marikova J., Kunes J. (2018). Scoulerine affects microtubule structure, inhibits proliferation, arrests cell cycle and thus culminates in the apoptotic death of cancer cells. Sci. Rep..

[89] Xie J., Xu Y., Huang X., Chen Y., Fu J., Xi M., Wang L. (2015). Berberine-induced apoptosis in human breast cancer cells is mediated by reactive oxygen species generation and mitochondrial-related apoptotic pathway. Tumor Biol..

[90] Wang Y., Li Y.-J., Huang X.-H., Zheng C.-C., Yin X.-F., Li B., He Q.-Y. (2018). Liensinine perchlorate inhibits colorectal cancer tumorigenesis by inducing mitochondrial dysfunction and apoptosis. Food Funct..

[91] Shukla S., Rizvi F., Raisuddin S., Kakkar P. (2014). FoxO proteins′ nuclear retention and BH3-only protein Bim induction evoke mitochondrial dysfunction-mediated apoptosis in berberine-treated HepG2 cells. Free Radic. Biol. Med..

[92] Xu F., Li Q., Wang Z., Cao X. (2019). Sinomenine inhibits proliferation, migration, invasion and promotes apoptosis of prostate cancer cells by regulation of miR-23a. Biomed. Pharmacother..

[93] Liu C., Gong K., Mao X., Li W. (2011). Tetrandrine induces apoptosis by activating reactive oxygen species and repressing Akt activity in human hepatocellular carcinoma. Int. J. Cancer.

[94] Kim S.Y., Hwangbo H., Lee H., Park C., Kim G.-Y., Moon S.-K., Yun S.J., Kim W.-J., Cheong J., Choi Y.H. (2020). Induction of apoptosis by coptisine in Hep3B hepatocellular carcinoma cells through activation of the ROS-mediated JNK signaling pathway. Int. J. Mol. Sci..

[95] Han B., Jiang P., Li Z., Yü Y., Huang T., Ye X., Li X. (2018). Coptisine-induced apoptosis in human colon cancer cells (HCT-116) is mediated by PI3K/Akt and mitochondrial-associated apoptotic pathway. Phytomedicine.

[96] Tian J., Mo J., Xu L., Zhang R., Qiao Y., Liu B., Jiang L., Ma S., Shi G. (2020). Scoulerine promotes cell viability reduction and apoptosis by activating ROS-dependent endoplasmic reticulum stress in colorectal cancer cells. Chem. Biol. Interact..

[97] Chen X.-M., Zhang M., Fan P.-L., Qin Y.-H., Zhao H.-W. (2016). Chelerythrine chloride induces apoptosis in renal cancer HEK-293 and SW-839 cell lines. Oncol. Lett..

[98] Kim J.-S., Oh D., Yim M.-J., Park J.-J., Kang K.-R., Cho I.-A., Moon S.-M., Oh J.-S., You J.-S., Kim C.-S. (2015). Berberine induces FasL-related apoptosis through p38 activation in KB human oral cancer cells. Oncol. Rep..

[99] Si Y., Wang J., Liu X., Zhou T., Xiang Y., Zhang T., Wang X., Feng T., Xu L., Yu Q. (2020). Ethoxysanguinarine, a Novel Direct Activator of AMP-Activated Protein Kinase, Induces Autophagy and Exhibits Therapeutic Potential in Breast Cancer Cells. Front. Pharmacol..

[100] Yu R., Zhang Z.-Q., Wang B., Jiang H.-X., Cheng L., Shen L.-M. (2014). Berberine-induced apoptotic and autophagic death of HepG2 cells requires AMPK activation. Cancer Cell Int..

[101] Stiborová M., Šimánek V., Frei E., Hobza P., Ulrichová J. (2002). DNA adduct formation from quaternary benzo [c] phenanthridine alkaloids sanguinarine and chelerythrine as revealed by the 32P-postlabeling technique. Chem. Biol. Interact..

[102] Lee Y.-K., Lee K.W., Kim M., Lee Y., Yoo J., Hwangbo C., Park K.H., Kim K.D. (2019). Chelidonine induces caspase-dependent and caspase-independent cell death through G2/M arrest in the T98G human glioblastoma cell line. Evid. Based Complement. Altern. Med..

[103] Chen C.-H., Liao C.-H., Chang Y.-L., Guh J.-H., Pan S.-L., Teng C.-M. (2012). Protopine, a novel microtubule-stabilizing agent, causes mitotic arrest and apoptotic cell death in human hormone-refractory prostate cancer cell lines. Cancer Lett..

[104] Jordan M.A., Wilson L. (2004). Microtubules as a target for anticancer drugs. Nat. Rev. Cancer.

[105] Lopus M., Panda D. (2006). The benzophenanthridine alkaloid sanguinarine perturbs microtubule assembly dynamics through tubulin binding: A possible mechanism for its antiproliferative activity. FEBS J..

[106] Cheriyamundath S., Mahaddalkar T., Nagireddy P.K.R., Sridhar B., Kantevari S., Lopus M. (2019). Insights into the structure and tubulin-targeted anticancer potential of N-(3-bromobenzyl) noscapine. Pharmacol. Rep..

[107] Panzer A., Joubert A.M., Bianchi P.C., Hamel E., Seegers J.C. (2001). The effects of chelidonine on tubulin polymerisation, cell cycle progression and selected signal transmission pathways. Eur. J. Cell Biol..

[108] Goldbrunner M., Loidl G., Polossek T., Mannschreck A., von Angerer E. (1997). Inhibition of tubulin polymerization by 5, 6-dihydroindolo [2, 1-a] isoquinoline derivatives. J. Med. Chem..

[109] Kim S.A., Kwon Y., Kim J.H., Muller M.T., Chung I.K. (1998). Induction of topoisomerase II-mediated DNA cleavage by a protoberberine alkaloid, berberrubine. Biochemistry.

[110] Kumar A., Chowdhury S.R., Sarkar T., Chakrabarti T., Majumder H.K., Jha T., Mukhopadhyay S. (2016). A new bisbenzylisoquinoline alkaloid isolated from Thalictrum foliolosum, as a potent inhibitor of DNA topoisomerase IB of Leishmania donovani. Fitoterapia.

[111] Kazemi Noureini S., Fatemi L., Wink M. (2018). Telomere shortening in breast cancer cells (MCF7) under treatment with low doses of the benzylisoquinoline alkaloid chelidonine. PLoS ONE.

[112] Ma Y., Ou T.-M., Tan J.-H., Hou J.-Q., Huang S.-L., Gu L.-Q., Huang Z.-S. (2009). Synthesis and evaluation of 9-O-substituted berberine derivatives containing aza-aromatic terminal group as highly selective telomeric G-quadruplex stabilizing ligands. Bioorganic Med. Chem. Lett..

[113] Zhang W.-J., Ou T.-M., Lu Y.-J., Huang Y.-Y., Wu W.-B., Huang Z.-S., Zhou J.-L., Wong K.-Y., Gu L.-Q. (2007). 9-Substituted berberine derivatives as G-quadruplex stabilizing ligands in telomeric DNA. Bioorganic Med. Chem..

[114] Wu H.L., Hsu C.Y., Liu W.H., Yung B.Y.M. (1999). Berberine-induced apoptosis of human leukemia HL-60 cells is associated with down-regulation of nucleophosmin/B23 and telomerase activity. Int. J. Cancer.

[115] Chan S.-L., Lee M.C., Tan K.O., Yang L.-K., Lee A.S.Y., Flotow H., Fu N.Y., Butler M.S., Soejarto D.D., Buss A.D. (2003). Identification of chelerythrine as an inhibitor of BclXL function. J. Biol. Chem..

[116] Lauf P.K., Heiny J., Meller J., Lepera M.A., Koikov L., Alter G.M., Brown T.L., Adragna N.C. (2013). Canonical Bcl-2 motifs of the Na+/K+ pump revealed by the BH3 mimetic chelerythrine: Early signal transducers of apoptosis?. Cell. Physiol. Biochem..

[117] Zhang X.J., Yang L., Zhao Q., Caen J.P., He H.Y., Jin Q.H., Guo L.H., Alemany M., Zhang L.Y., Shi Y. (2002). Induction of acetylcholinesterase expression during apoptosis in various cell types. Cell Death Differ..

[118] Giacobini E. (2003). Cholinergic function and Alzheimer′s disease. Int. J. Geriatr. Psychiatry.

[119] Greig N.H., Utsuki T., Yu Q.S., Zhu X., Holloway H.W., Perry T., Lee B., Ingram D.K., Lahiri D.K. (2001). A new therapeutic target in Alzheimer’s disease treatment: Attention to butyrylcholinesterase. Curr. Med. Res. Opin..

[120] Morgan H.D., Santos F., Green K., Dean W., Reik W. (2005). Epigenetic reprogramming in mammals. Hum. Mol. Genet..

[121] Feinberg A.P., Ohlsson R., Henikoff S. (2006). The epigenetic progenitor origin of human cancer. Nat. Rev. Genet..

[122] Jones P.A., Baylin S.B. (2002). The fundamental role of epigenetic events in cancer. Nat. Rev. Genet..

[123] Esteller M. (2007). Cancer epigenomics: DNA methylomes and histone-modification maps. Nat. Rev. Genet..

[124] Allis C.D., Jenuwein T. (2016). The molecular hallmarks of epigenetic control. Nat. Rev. Genet..

[125] Riggs M., Whittaker R.G., Neumann J.R., Ingram V.M. (1977). n-Butyrate causes histone modification in HeLa and Friend erythroleukaemia cells. Nature.

[126] Berghe W.V. (2012). Epigenetic impact of dietary polyphenols in cancer chemoprevention: Lifelong remodeling of our epigenomes. Pharmacol. Res..

[127] Zhang L., Miao X.-J., Wang X., Pan H.-H., Li P., Ren H., Jia Y.-R., Lu C., Wang H.-B., Yuan L. (2016). Antiproliferation of berberine is mediated by epigenetic modification of constitutive androstane receptor (CAR) metabolic pathway in hepatoma cells. Sci. Rep..

[128] Kalaiarasi A., Anusha C., Sankar R., Rajasekaran S., Marshal J.J., Muthusamy K., Ravikumar V. (2016). Plant isoquinoline alkaloid berberine exhibits chromatin remodeling by modulation of histone deacetylase to induce growth arrest and apoptosis in the A549 cell line. J. Agric. Food Chem..

[129] Hu H.-Y., Li K.-P., Wang X.-J., Liu Y., Lu Z.-G., Dong R.-H., Guo H.-B., Zhang M.-X. (2013). Set9, NF-κB, and microRNA-21 mediate berberine-induced apoptosis of human multiple myeloma cells. Acta Pharmacol. Sin..

[130] Huang C., Liu H., Gong X.-L., Wu L.-Y., Wen B. (2017). Effect of evodiamine and berberine on the interaction between DNMTs and target microRNAs during malignant transformation of the coln by TGF-β1. Oncol. Rep..

[131] Selvi R., Pradhan S.K., Shandilya J., Das C., Sailaja B.S., Gadad S.S., Reddy A., Dasgupta D., Kundu T.K. (2009). Sanguinarine interacts with chromatin, modulates epigenetic modifications, and transcription in the context of chromatin. Chem. Biol..

[132] Qing Y., Hu H., Liu Y., Feng T., Meng W., Jiang L., Sun Y., Yao Y. (2014). Berberine induces apoptosis in human multiple myeloma cell line U266 through hypomethylation of p53 promoter. Cell Biol. Int..

